# Appraising Mahallat Geothermal Region using thermal surveying data accompanied by the geological, geochemical and gravity analyses

**DOI:** 10.1038/s41598-021-90866-4

**Published:** 2021-06-09

**Authors:** Javad Nouraliee, Davar Ebrahimi, Ali Dashti, Maziar Gholami Korzani, Sepehr Sangin

**Affiliations:** 1 Renewable Energy Department, Energy and Environmental Research Center, Niroo Research Institute, Tehran, Iran; 2 Institute of Applied Geosciences, Karlsruhe Institute of Technology, Adenauerring 20b, 76131 Karlsruhe, Germany; 3 University of Göttingen, Göttingen, Germany

**Keywords:** Geothermal energy, Geology

## Abstract

Mahallat Geothermal Region, located in the central part of Iran, is known as one of the largest low-temperature geothermal fields. In this study, Mahallat geothermal resource has been evaluated based on integrated geological, geochemical and geophysical analyses. Gravity data revealed three major negative anomaly zones. Based on the geochemical analyses, quartz geothermometers are more reliable than others and confirmed that the reservoir is about 90 °C. Lithological properties of Jurassic layers and high sulphate content observed in geochemical data showed traces of the coal-rich layers on the hot fluids. Measured temperatures in 7 boreholes with the depths ranging from 50 to 100 m, have proposed that expected geothermal gradient will be about 81.5 °C/km. Among all drilled boreholes, the data coming from only one resulted in this almost reliable gradient. Other boreholes are clearly too shallow or affected by upflow or downflow of water along existing faults. Geological, geochemical, gravity and measurements of drilled boreholes suggested the existence of a shallow reservoir with an approximate temperature of 90 °C. Regarding gravity and observed faults, geothermal reservoir is elongated parallel to one of the main faults of the region with NE-SW strike.

## Introduction

Energy is an influential factor for macroeconomic growth, prosperity and development of society. Energy supply is considered as an important element of sustainable development^1^. Geology acts as a controlling factor in the formation and distribution of a renewable source of energy (geothermal resources)^2^. Geological field exploration and tectonic studies^2–4^, hydrological analyses^5^, geochemical sampling and testing^5–7^, geophysical methods^8–10^, measurements in geothermal wells^11^, airborne methods^12^ and satellite data^13^ are some of practical tools for evaluating the potential of a geothermal area. Mentioned methods and tools can show different characteristics of a geothermal prospect. After exploration, a wide variety of methods can be exerted to model and simulate the hot underground reservoir. Temperature, pressure, flow regime, fracture network are known as the most important factors^14–18^.

In the most simplified cases, a geothermal reservoir can be explored merely because of observable surface evidence such as hot springs. But, sophisticated exploration procedures are required to make a reliable prediction on the geothermal potential of a prospect. Exploration of the geothermal resources in Iran (world's second and fourth largest reserves of gas and oil, respectively^19,20^) can be regarded as an unreasonable task due to the availability and cheapness of the fossil fuels. A detailed history of geothermal explorations in Iran since 1975 is available in the literature^21,22^. Installation of the first Iranian geothermal power plant at the Sabalan Field (northwest of Iran) is the result of some country-wide exploration projects. In addition, further detailed geothermal exploration studies have been carried out in different parts of Iran^17–25,26^.

Mahallat is also another possible geothermal site located in the Markazi Province of Iran. Three negative gravity anomaly zones and faults (acting as preferential pathways for hydrothermal fluids circulation) are confirmed in this area^21–29^. Published documents mainly presented all interpretations on the Mahalat geothermal region merely based on the geophysical data. But, geochemical and gradient wells can be used to bring more integrated interpretations for the underground reservoir^11^.

An integrated exploration scheme of Mahallat Geothermal Region is the main contribution of the presented study. To do so, it is attempted to understand features such as heat source, hydrothermal fluids properties, and probable pathways of fluids using an almost complete data set. Measurements carried out in 7 boreholes are analyzed to find out the geothermal gradient of Mahallat Geothermal Region.

## Geological setting

**Figure 1:**
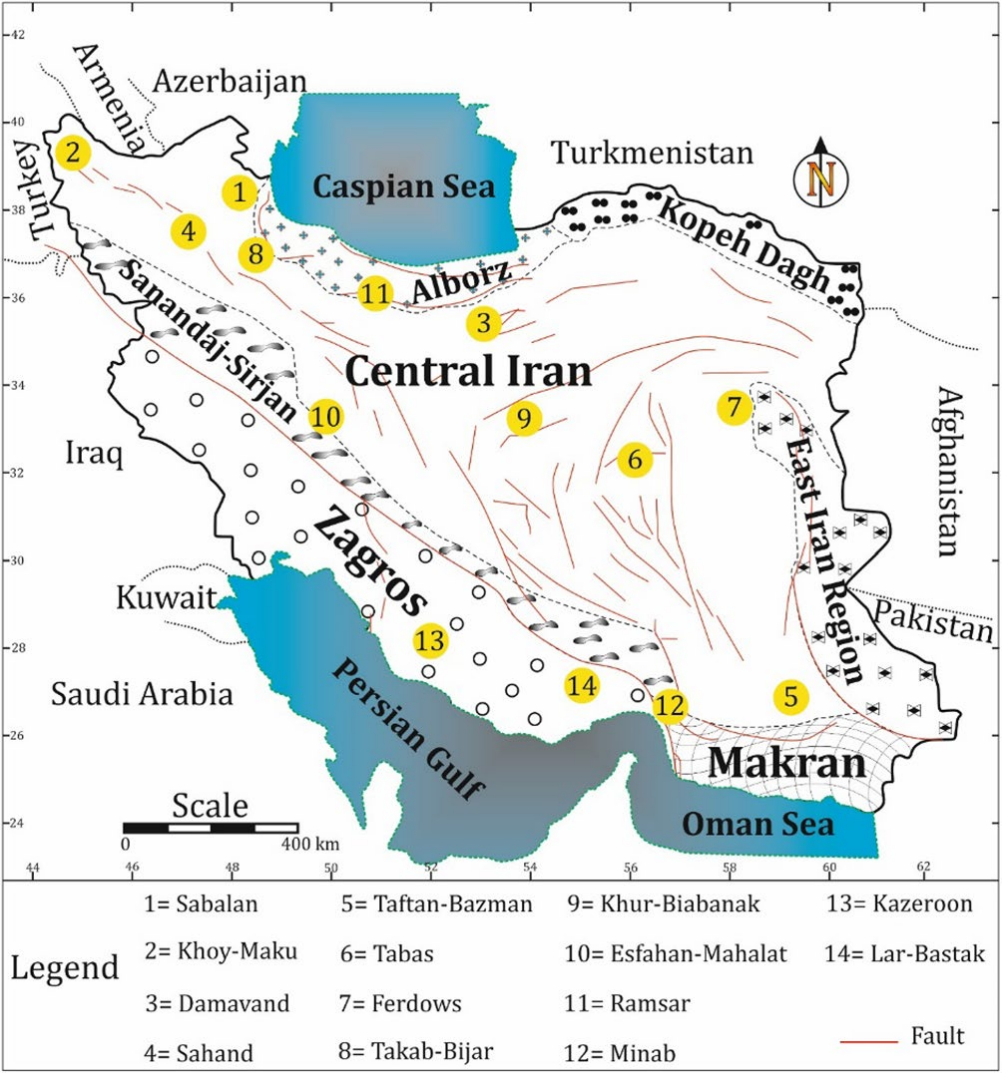
Geologic zonation of Iran and geothermal prospects existing within this plateau (adopted from^22,40^ and generated by corelDRAW version 22.1.1.523). Numbers represent the location and each location with its related number is explained in the legend. Numbers also show the importance of locations decreasing from 1 to 14.

**Figure 2:**
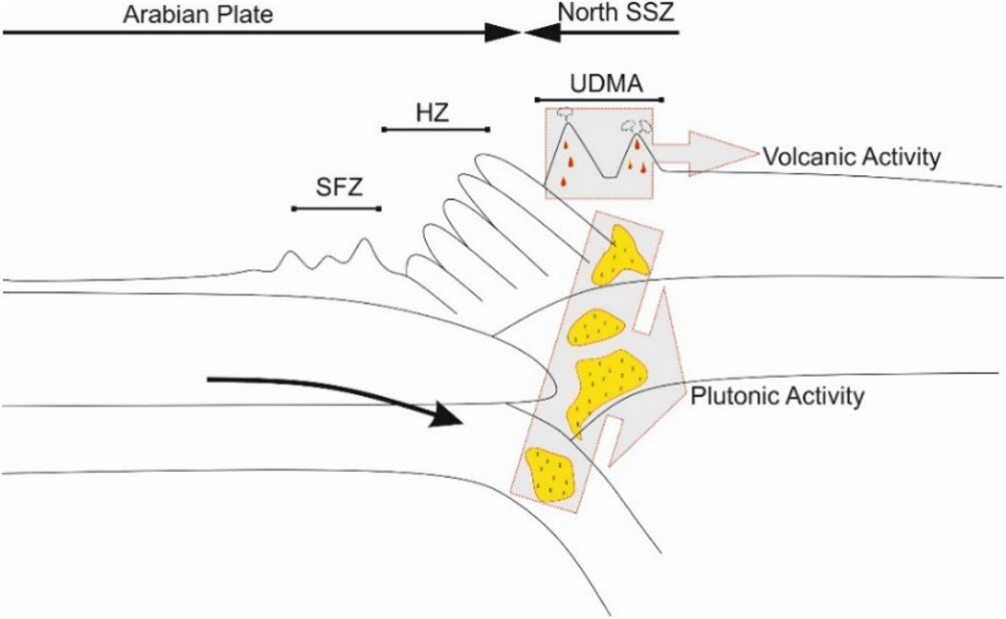
Tectonic evolution of the North Sanandaj–Sirjan Zone (NSSZ) during Middle Eocene (adopted from^38^). HZ and SFZ stand for High Zagros and Simply Folded Zagros, respectively.

**Figure 3:**
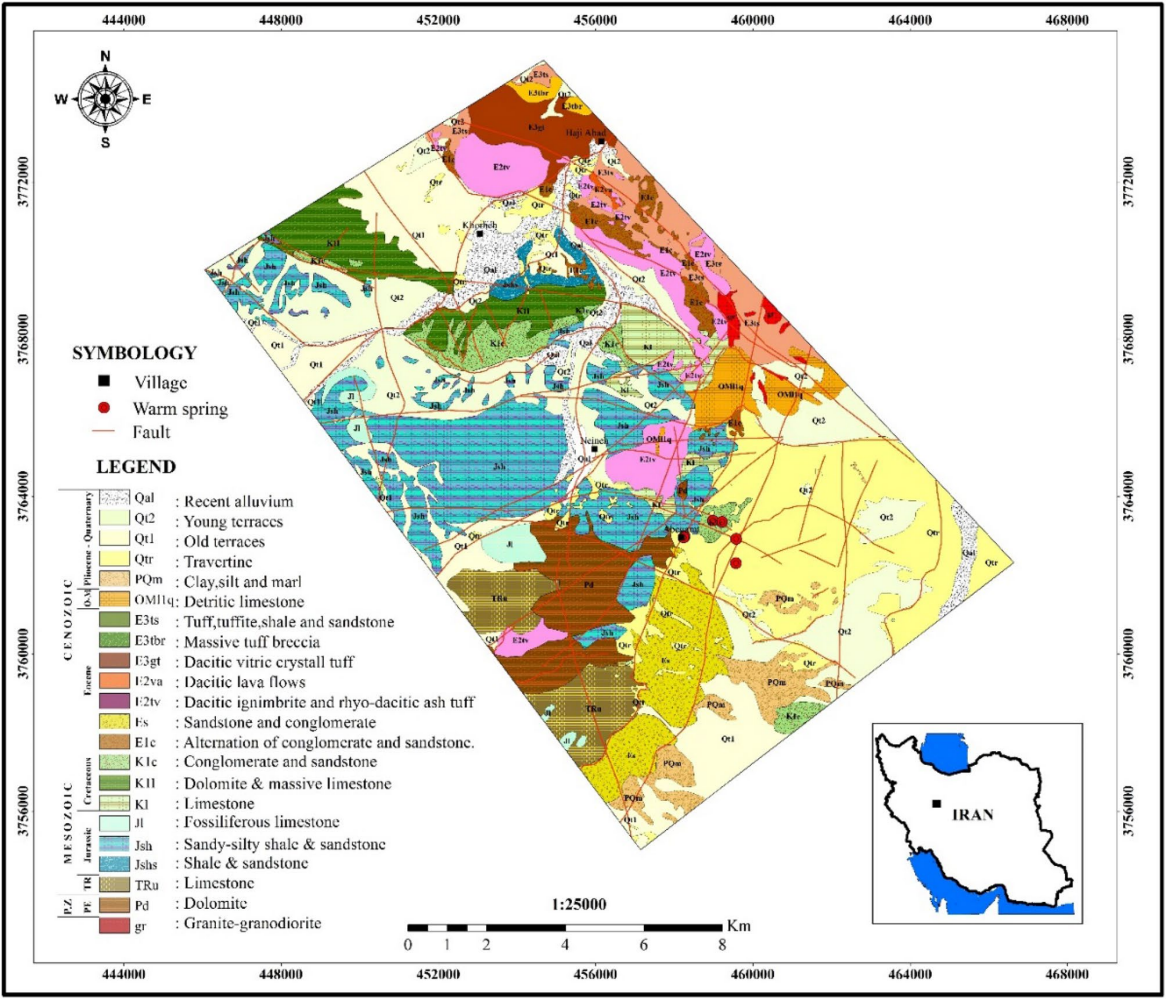
Detailed Geological map of the Mahallat Region (with 1:25,000 scale).

Studied region (Mahallat) is geographically located in the central Iran. From a geologic point of view, studied area belongs to geologic Sanandaj-Sirjan Zone (SSZ) (the NW-SE trending zone in the north of Zagros Region) (Fig. 1). This zone is composed of Mesozoic and rarely Paleozoic metamorphosed and deformed units. As discussed in the literature^30,31^, geology of Iranian Plateau is complicated and different scenarios are presented to bring reliable interpretations. The adjacency of oil-rich sedimentary basins of Zagros Region and geothermal prospects of SSZ is an evidence confirming the geological complexity of the Iranian plateau. Subduction of Neo-Tethys beneath Central Iranian microcontinent can be the source of this complexity. Researchers like^32,33^ suggested that the subduction started during late Triassic—early Jurassic time, while^34,35^ proposed the early to late Cretaceous as the start. Stern^36^ has discussed all the details of the subduction process and outcomes. Magmatism of Urumieh–Dokhtar Magmatic Assemblage (UDMA) started Early Eocene and continued until Pleistocene^37^. The most volcanic active period of UDMA is found to be in the Middle to Upper Eocene^34^. Figure 2 (extracted from^38^) schematically shows a summarized and simple illustration on the complicated processes that formed the geology of studied area. The figure is presenting the situation of the studied area in the middle Eocene. Subduction of Arabia under Iranian Plateau resulted in the formation of plutonic and volcanic masses within SSZ and UDMA. Mahallat geothermal prospect is formed in a convective setting by the adjacency of magmatic intrusions. As Fig. 2 shows, plutonic masses are an inevitable consequent of subduction. Numerous dextral strike-slip faults (caused by convergence) are acting as the major pathway for circulating fluids from the surface toward deep hot masses^39^. In the Mahallat Region, existence of a complex fault system is confirmed based on the horizontal gradient maps^29^. Merely based on the discussed publications, existence of a complicated fault system can be well imagined in the study area. The detailed 1:25,000 geological map of the study area is shown in Fig. 3. There are some geological and geothermal evidence suggesting a probable geothermal resource in Mahallat Region. Outcrops of travertine and granitic rocks, several warm springs and long faults are some of the observable evidence. Due to the lack of young volcanic rocks in the region, the probable heat source of the geothermal resource can be radioactive decay of the extensive granitic rocks. As geologic map shows, a diverse set of sedimentary rocks including limestones, sandstones and with lower abundance, shale, conglomerate and marlstones are covering the area. There are only some volcanic rocks outcroping in NE and central part of the study area.

## Geochemistry studies

Geochemical studies are used to explore and exploite geothermal resources. Finding chemical composition of the geothermal fluid, geothermal reservoir temperature and flow direction are main goals of geochemical studies^41^. In this study, geochemical analyses of Mahallat Region have been carried out based on the chemical tests of samples coming from warm springs. These springs are located close to the Abgarm Village (shown in the geological map (Fig. 3)). Warm springs are considered as one of the most observable surface evidence of the underground hot reservoirs. Table 1 represents the general properties of these springs and the results of the geochemical analyses are summarized in Table 2.

**Table 1:** General characteristics of warm springs in Mahallat Region.

Spring Name	Longitude	Latitude	Altitude	Temperature (°C)	Discharge (liter per second (l/s))	pH	Electrical Conductivity (EC) (µs/Cm)
Ab Donbeh (AND)	459031	3763453	1848	46.7	1.45	6.45	9.02
Ab Soleymanieh (ASL)	459201	3763341	1826	46.3	–	6.55	9.8
Ab Soda (ABS)	459560	3762953	1737	49	–	6.54	10.005
Ab Hakim (AHK)	458919	3762289	1753	34.8	–	6.87	10.14
Shafa (SHF)	458344	3762921	1870	47.1	–	6.61	9.85

Temperature, discharge and electrical conductivity data are reported in degrees Celsius (°C), liter per second (l/s) and microSiemens per centimeter (µs/Cm), respectively.

**Table 2:** Results of geochemical analyses in Mahallat Region.

Geochemical Component (miligram/l)	Spring				
	Ab Soda	Ab Hakim	Ab Donbeh	Ab Soleymanieh	Shafa
NH_3_	0.005 >	0.005 >	0.005 >	0.005 >	0.005 >
Suspended solids	0.9	0.8	1.9	0.1	1.7
HCO^−^_3_	236.4	234.1	236.4	208.6	238.8
Mg^+^	72.7	67.8	72.7	82.4	72.7
Na^+^	100	95	95	105	95
K^+^	5.5	5.4	5.4	5.9	5.5
Cl^-^	43	44	45.9	51	45.9
SO^2−^_4_	1126	1135	1108	1182	1102
B^3+^	N.D (< 0.005)	0.1	0.03	0.08	0.08
Fe^2+^	0.7	0.2	0.65	0.02	0.76
SiO_2_	37.5	36	36	36	36
TDS (180 °C)	1126	1135	1108	1182	1102
Li^+^	0.4	0.4	0.4	0.4	0.4
S^2−^	N.D (< 0.1)	N.D (< 0.1)	N.D (< 0.1)	N.D (< 0.1)	N.D (< 0.1)
CO_2_	49.5	58	50	27.2	54
As^3−^	N.D (< 0.1)	N.D (< 0.1)	N.D (< 0.1)	N.D (< 0.1)	N.D (< 0.1)
F^−^	1	1	1	1.5	1
Al^3+^	< 0.02	< 0.02	< 0.02	< 0.02	< 0.02

### Cl-SO_4_-HCO_3_ diagram

**Figure 4:**
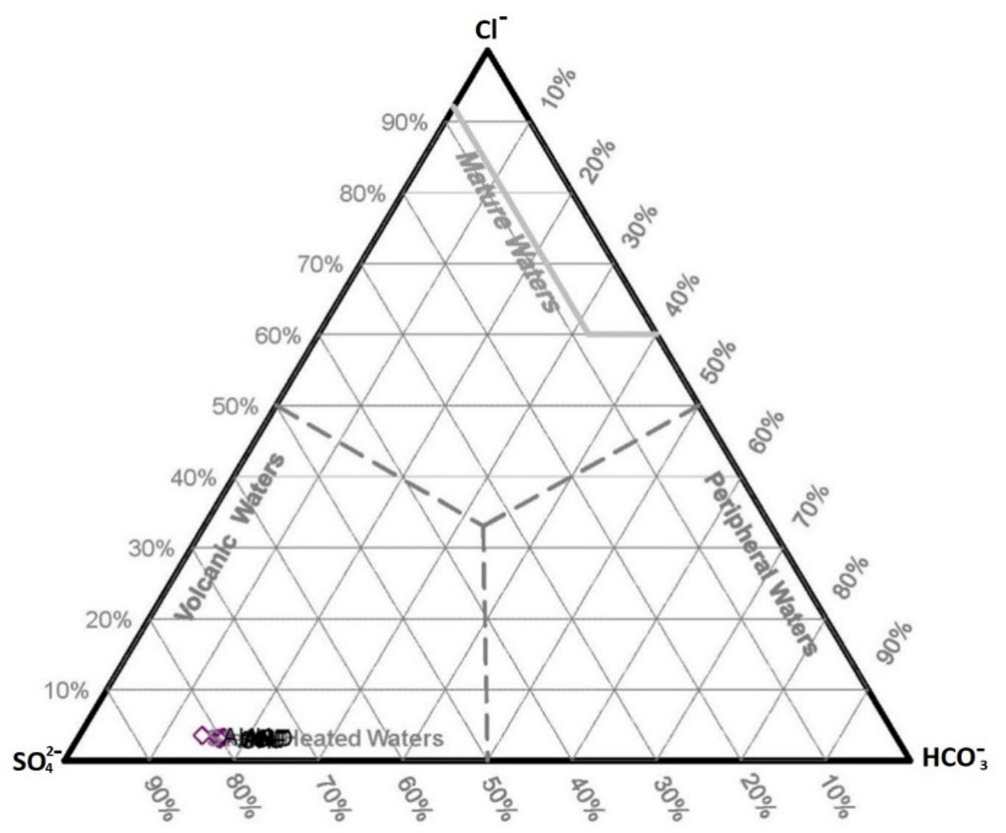
Cl-SO_4_-HCO_3_ diagram of hot springs in Mahallat Region.

**Figure 5:**
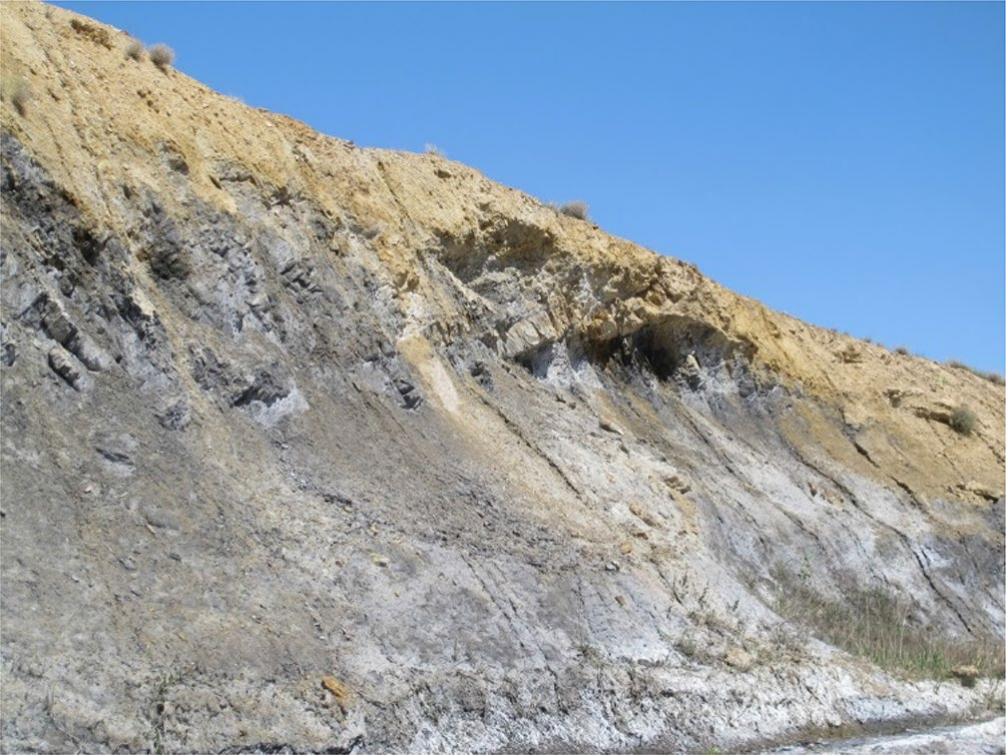
Coal rich layers of Shemshak Formation deposited in the Mahallat Geothermal Region (photo taken by one of the authors).

This diagram is mainly exerted to find the fluids' origin and classify the geothermal fluid^42^. Results of chemical analysis show all the samples gathered in the area fall into boiling water and are also located in the vicinity of the volcanic zone (Fig. 4). These fluids are formed when the geothermal fluids charge into underground waters. They usually contain HCl and H_2_S gases in high-temperature and low-temperature systems. Regarding available evidence, the source of flowing out fluids cannot be related to this type of reservoirs (volcanic zone). Meanwhile, geological map (Fig. 3) also clearly confirms that only a negligible part of the area is covered with volcanic rocks. The main reason for the relatively high concentration of HCl and H_2_S in these fluids can be related to the geology of the region, i.e. existence of sedimentary layers containing such elements (S and Cl). Chemical analyses revealed that the considerable concentration of sulphate ions can be due to the presence of coal layers observed in the Shemshak Formation. This Jurassic aged formation acts as the cap rock of reservoir (Fig. 5). High SO_4_ content in analyzed fluids can be the direct result of these coal layers. These hot fluids are highly susceptible for dissolving the sulphate ions deposited in coaly Jurassic layer. The geological map (Fig. 3) clearly shows that these Jurassic layers are covering a considerable part of the area. Dissolved S and Cl elements affect the interpretations on this diagram. In fact, they can be regarded as contaminations rather than native element of the geothermal fluids. As mentioned, it is almost impossible to encounter fluid with volcanic origin in the area, regarding the geology of Mahallat Region. It can be imagined reducing the amount of these two elements coming from coaly layers, shifts the data toward the bicarbonate corner of the ternary plot (HCO_3_) (bicarbonate water family). Abundance of coaly layers and lack of volcanic rocks both raise doubt about accuracy of the results can be obtained from Fig. 4.

### Na–K–Mg diagram

Na–K–Mg ternary diagram directly reveals the maturity of fluids and is widely used to evaluate reservoir temperature and also rock equilibrium. This diagram is divided into three subzones: immature, partial equilibrium and equilibrium water. It is a suitable tool to understand the degree of fluid-rock exchange. Mixing of geothermal fluids with groundwater increases the Mg content of the fluids and shifts them toward the immature water subdomain, i.e. close to the Mg corner.

**Figure 6:**
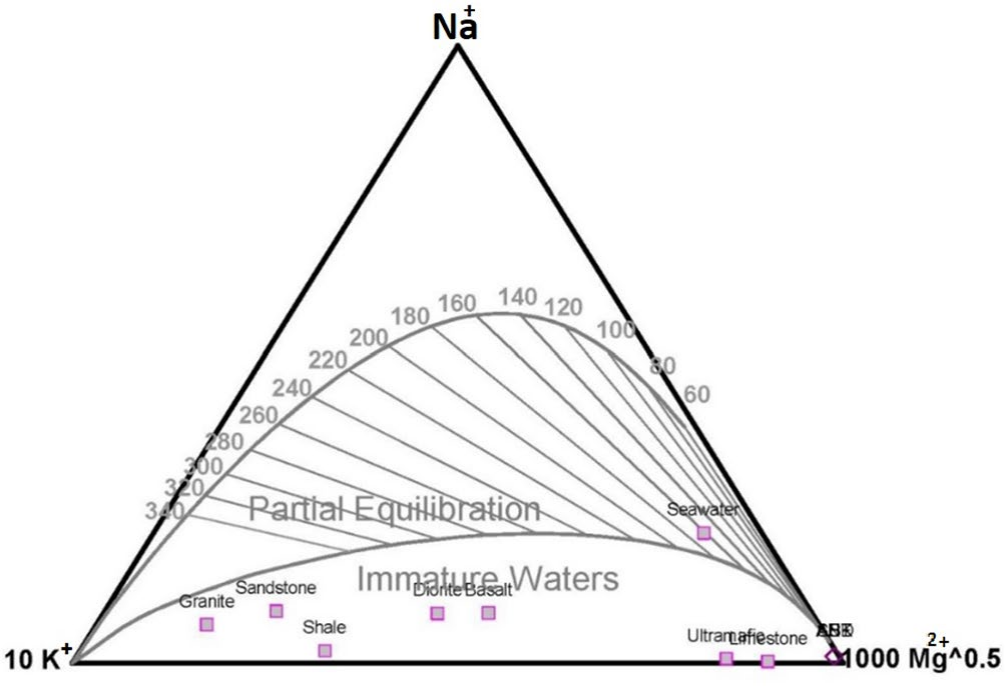
Na–K–Mg ternary diagram and distribution of hot springs' samples.

Samples of Mahallat Region are distributed around the Mg corner and belong to the boundary of the immature and partial equilibrium zone (Fig. 6). This immaturity implicates the short residence time of fluid in the hot reservoir. In other words, underground water is continuously flowing into geothermal fluids. This mixture is consequently decreasing the temperature of the Mahallat geothermal prospect. The reservoir temperature data (geothermometry section) can be an evidence to evaluate this interpretation.

### Geothermometry

Geothermometry of geothermal reservoirs are determined using fluids, steam and gas, and isotopes^43^. Calculations of underground temperature are usually carried out based on the concentration of elements like quartz, potassium, calcium and sodium^6,7,44^. A complete data set including geologic and geochemical data is highly important for selecting the most reliable geothermometers. In this study, cation geothermometers of Powell and Cumming, 2010 software is used to calculate the reservoir temperature. Calculated results are presented in Table 3. Considering the amount of quartz in the results of geochemical analyses (Table 2), quartz geothermometers are more reliable than others. On the basis of quartz geothermometer, temperature of the Mahallat Geothermal Reservoir is estimated to be 90 °C.

**Table 3:** Results of different geothermometric methods in studied locations [temperatures are reported in degrees celsius (°C)].

Spring	Chalcedony cond	Quartz cond	Quartz adiabatic	K/Mg (Giggenbach)	Na/K (Giggenbach)	Na/K Truesdell	Na/K Fournier	Na–K–Ca
SHF	58	89	91	35	189	131	171	1
ASL	56	88	90	35	191	134	173	16
AND	56	88	90	34	191	134	173	2
AHK	56	88	90	35	190	133	172	− 16
ABS	56	88	90	35	192	192	171	1

## Geophysical studies

Two different types of the geophysical surveys have been conducted to present a more integrated interpretation on the studied prospect. In the presented study, these two types are gravity and thermal surveying.

### Gravity surveying

**Figure 7:**
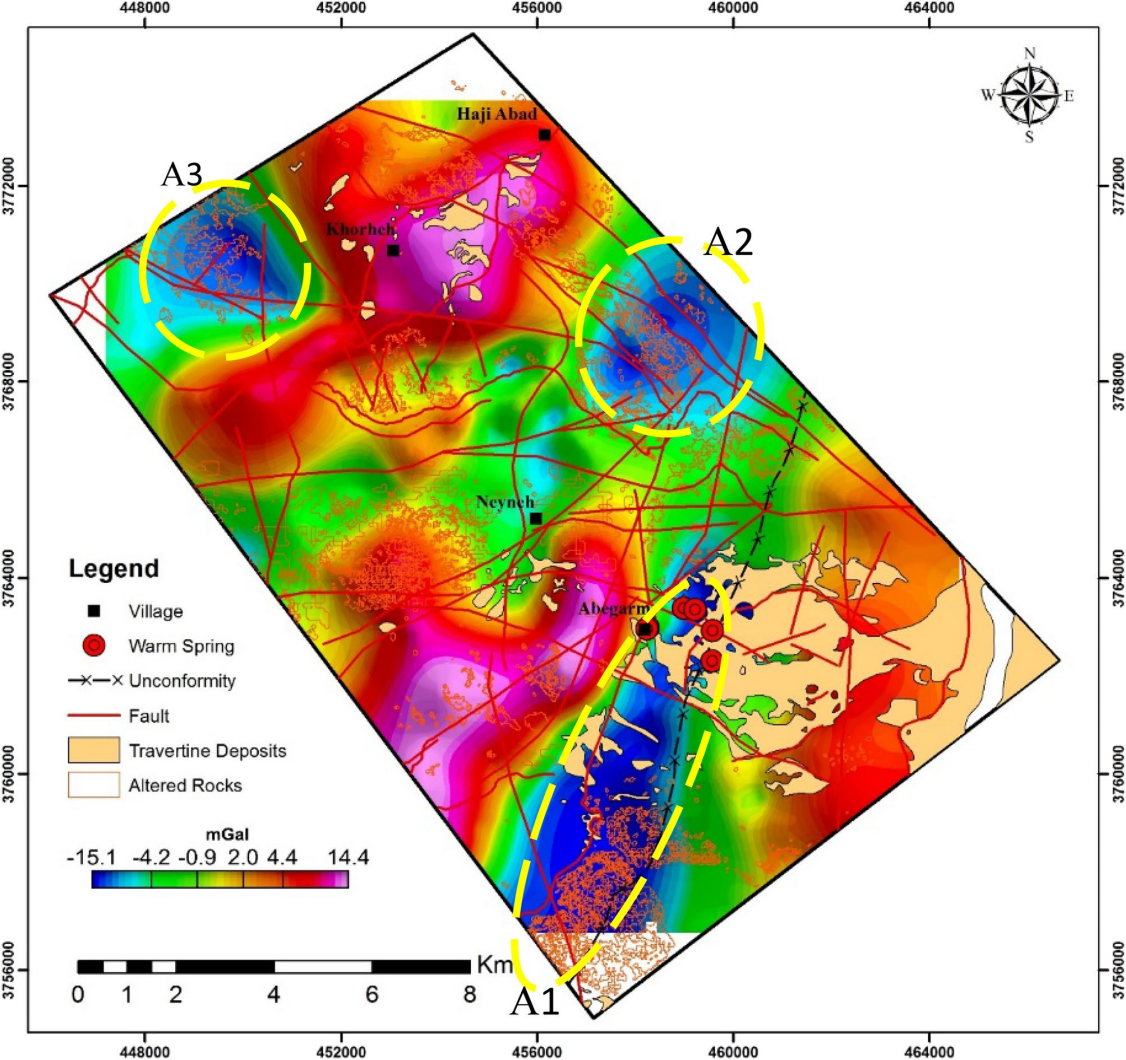
Residual gravity anomaly map of Mahallat Region a surface manifestation of geothermal resources including warm springs, travertine deposits and altered rocks (figure exported from GEOSOFT Oasis montaj 9.2 available in https://www.seequent.com).

Gravity data are exerted to identify a range of geological structures related to magmatic intrusion into host rock, caldera collapse, depth of basement rock, or faults^45,46^. Gravity data of Mahallat Geothermal Region were collected by a Scintrex CG5 gravimeter in 380 stations covering an area of approximately 220 km^2^. Figure 7 shows the complete Bouguer anomaly map of the region. Distances between stations are not equal and vary from 200 to 450 m due to the topography and the accessibility of the terrain.

As a result of gravity studies, three negative anomaly zones were determined in the region. These zones are referred as A1, A2 and A3 in Fig. 7. In this part of Mahallat Region, there are five warm springs indicating an active geothermal resource in deep parts of the region. It should be noted that in Mahallat Region warm springs exit only in A1 anomaly i.e. warm springs are located above the A1 anomaly. Moreover, in A1 anomaly there are several extensive travertine outcrops showing the relatively long time activity of warm springs in this zone. Argillic, propyllitic and the Fe-oxide altered rocks are observed in the northernmost and southernmost parts of A1 anomaly. They might be related to the present or previous activities of a geothermal system in this region. A1 anomaly is surrounded by two different geological phenomena in the northern and southern sides. North Mahallat unconformity with 5 km length is observed in the south side of A1 anomaly and makes the eastern border. In the west side, a thrust fault called Abgarm Fault with 10 km length is observed. Its strike is NE-SW and its dip is to the northwest direction. This fault moved Triassic carbonate units over Eocene conglomerate sandstone units. Abgarm Fault has a distinctive role in the formation of the geothermal reservoir in Mahallat Region. A2 and A3 gravity anomalies have much lower importance from geothermal point of view. Regarding surface geothermal manifestations of these two zones, they are les active than A1. There is only weak evidence of Fe-oxide and Propyllitic altered rocks in A2 and it lacks any signature of geothermal activity at the surface. Abgarm thrust fault passes through this anomaly too. A2 anomaly is formed as an ellipsoid which its large-diameter as about 5 km. A3, in an ellipsoid from, anomaly is located in the west of Khorheh Village. The larger diameter of this ellipsoid is about 4 km and it is elongated in the NW-SE direction. Some propyllitic altered rocks are also observed in A3 anomaly zone. These altered rocks are the only surface manifestation of a geothermal resource in this anomaly.

### Geothermal survey

From the surface toward the center of the Earth temperature increases but the accurate rate of increase is unknown. Documents are published to present an understanding of temperature changes within different fields^41–50^. Understanding the geothermal gradient is notably important for hot reservoirs exploration and underground heat harnessing. To find out the geothermal gradient drilling of gradient wells is regarded as an efficient method^11,51,52^.

Considering previous studies in this area, 15 drilling locations were suggested but after implementation of gravity measurements, 7 locations were finally chosen. However, it is worthwhile mentioning easier access routes to the drilling sites has also influenced the decision-making process of selecting the appropriate location. Table 4 represents the name, location and depth of drilled thermal gradient wells. Drilling difficulties prevented reaching to the desired depth (90 to 100 m) in three boreholes (BH-04, BH-05 and BH-06).

**Table 4:** General properties of drilled wellbores in Mahallat Region.

Borehole	Longitude	Latitude	Depth (m)
BH-01	50° 33′ 01″	34° 00′ 30″	100
BH-02	50° 35′ 11″	34° 01′ 43″	90
BH-03	50° 33′ 22″	34° 00′ 14″	102
BH-04	50° 32′ 08″	33° 58′ 06″	50
BH-05	50° 32′ 00″	33° 59′ 19″	50
BH-06	50° 32′ 51″	33° 59′ 05″	50
BH-07	50° 32′ 45″	34° 00′ 13″	100

**Figure 8:**
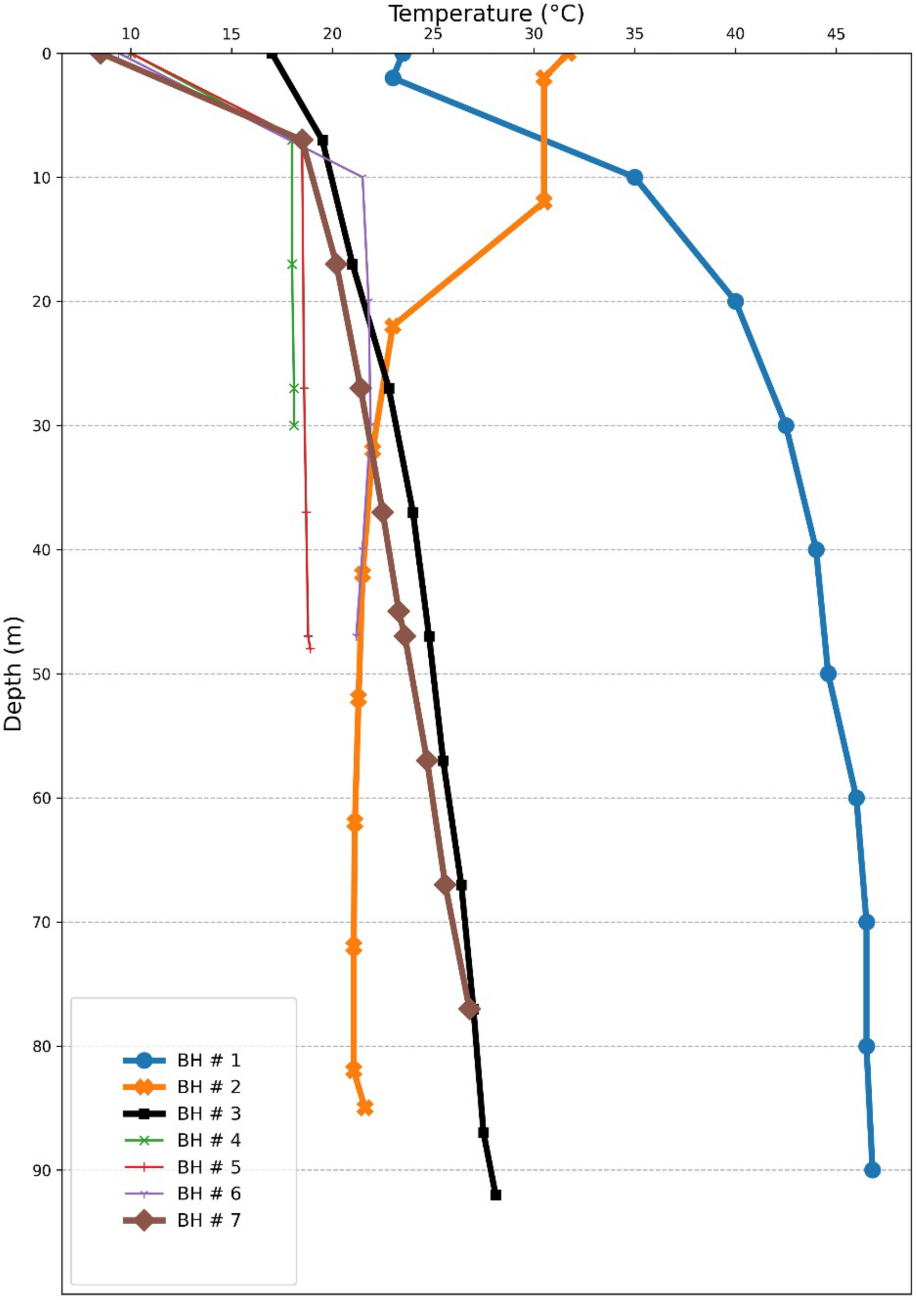
Temperature changes versus depth profile of the wells.

After drilling the wells, temperature is recorded at depths of 7 boreholes. Results of measurements are presented in Fig. 8. As mentioned, the drilling was almost failed in some of the boreholes. Another difficulty was measuring the temperature. In some boreholes the measurement tool stuck in some depths and it was impossible to do the measurment further. For example, BH-04 can be considered as a failed case, because its final depth is 50 m and measurement was only possible for the first 30 m. These difficulties have been predicted beforehand and 7 locations have been selected to overcome the problems and yield reliable results. Regarding the temperature versus depth diagram of all wells (Fig. 8), anomalies in temperature change (notable increase or decrease) through depth are observed in BHs # 1, #2, #3, and #7. These four boreholes are shown by thicker lines and bigger markers in Fig. 8. Table 5 also represents all the measured temperatures of the drilled boreholes.

**Table 5:** Measured temperature in 7 boreholes.

Borehole	Depth (m)	Temperature (°C)
BH-1	0	23.5
	2	23
	10	35
	20	40
	30	42.5
	40	44
	50	44.6
	60	46
	70	46.5
	80	46.5
	90	46.8
BH-2	0	31.7
	2	30.5
	12	30.5
	22	23
	32	22
	42	21.5
	52	21.3
	62	21.12
	72	21.06
	82	21.06
	85	21.62
BH-3	0	17
	7	19.5
	17	21
	27	22.8
	37	24
	47	24.8
	57	25.5
	67	26.4
	77	27
	87	27.5
	92	28.1
BH-4	0	10
	7	18
	17	18
	27	18.1
	30	18.1
BH-5	0	10
	7	18.5
	27	18.6
	37	18.7
	47	18.8
	48	18.9
BH-6	0	9.4
	10	21.5
	20	21.8
	30	21.9
	40	21.5
	47	21.188
BH-7	0	8.5
	7	18.5
	17	20.2
	27	21.4
	37	22.5
	45	23.3
	47	23.6
	57	24.7
	67	25.6
	77	26.8

In BH #2 the temperature is decreasing that could be resulted by the inflow of groundwater. This mixture is also observed in the geochemical data and the plot exerted for maturity evaluation (Fig. 6). Clearly, measurements coming out of this borehole are unable to yield reliable results to calculate geothermal gradient. Considering the geothermal surface manifestations in the vicinity of the BHs #1, #3 and #7, this area can be considered as upward flow zone of geothermal fluid. Therefore, studying the measured temperature gradients of these 3 wells may give more accurate results on geothermal gradient values in Mahallat Region. But, BH #1 is also clearly representing the convection phenomena. In a very short distance, the temperature has an abrupt increase implicating the convection cells. Therefore, only BH #3 and BH #7 can be used to calculate the geothermal gradient.

Thermal gradients were calculated for 3 gradient wells based on the gradient equation:

1 $$GT = \frac{{T_{depth} - T_{surface} }}{Depth}$$

**Figure 9:**
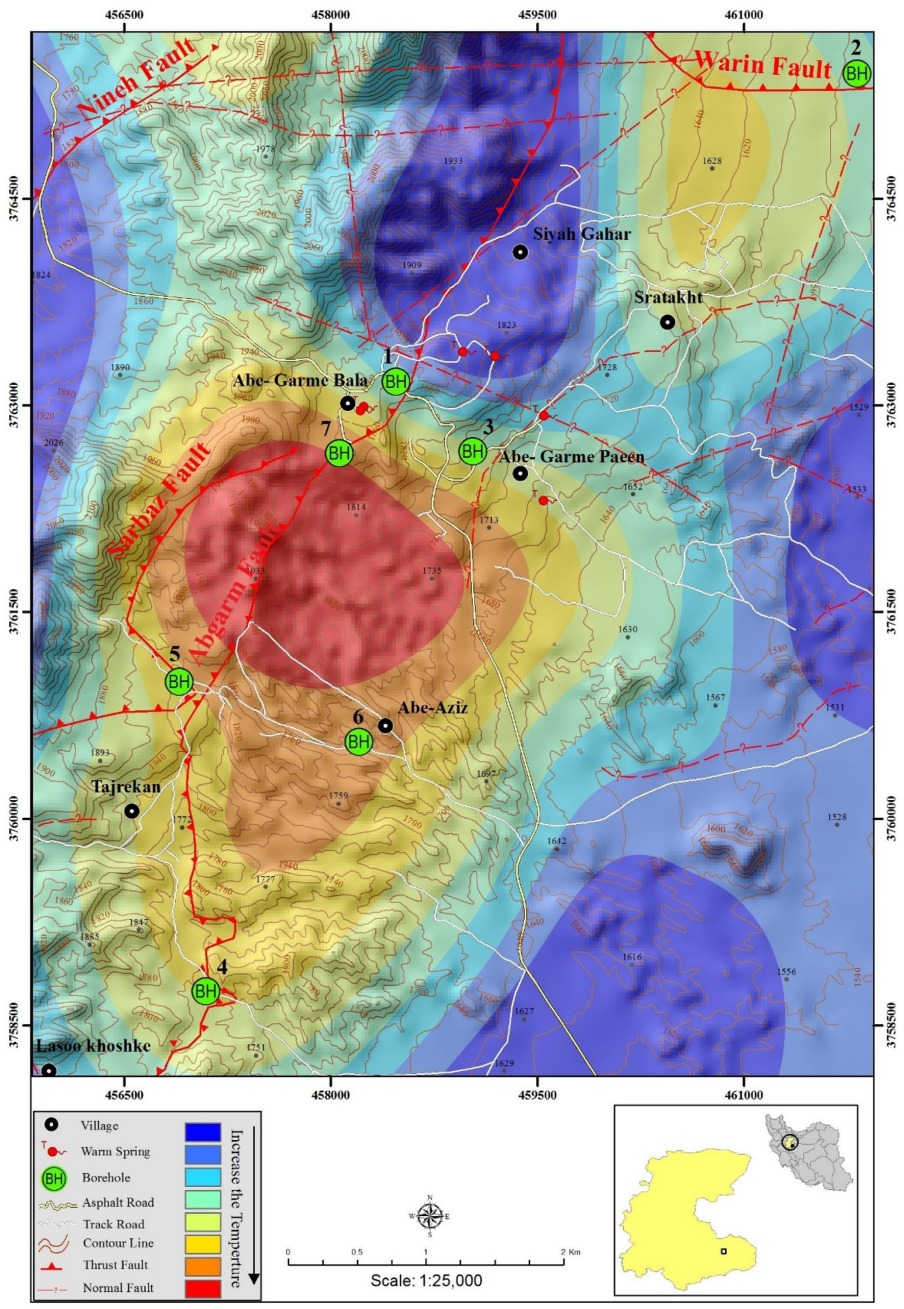
Temperature map at 50 m depth in the gradient wells (photo generated by ArcGIS 10.7.1).

In this study, gradient is calculated for the section that shows a conductive (linear) geothermal gradient. In BH # 3, it can be seen that after the third measurement in the depth of 17 m, the curve started to behave linearly. Therefore, the fourth and last measurements are used to measure the gradient in that interval. In this borehole, a 81.5 °C/km gradient is resulted. Due to the shallowness of boreholes, measured data are unable to provide an absolutely reliable gradient. Therefore, it is necessary to take a look into other available data. As geothemometery results revealed, the reservoir temperature is about 90 °C. Regarding the gradient, it can be expected to have such a reservoir in shallow depths (about 1 km). For the BH # 7, the curve starts to behave linearly in deeper parts. Fifth and last measurements are exerted to calculate the geothermal gradient in this borehole. Resulted value (107.5 °C/km) is surprisingly high in this borehole and needs to be discussed more. Adjacency of BH # 7 to the Abgarm Fault (visible in Fig. 9) confirms that hot fluid in this borehole is coming via a short circuit created by the fault. Therefore, regarding the geothermal gradient values in the region, the role of the faults in the formation of the geothermal reservoir is confirmed. Such hot fluids can only be carried out to the reservoir zone from the heat source through existing faults. Another fact is that this value is definitely exaggerated because of the shallowness of this borehole. For example, in Fig. 8 it is clear that the temperature profile of this borehole is reaching curve of BH # 3 at the depth of 77 m. After this depth, temperature of BH # 3 is almost stable which results in a less gradient in that borehole. But, in the case of BH # 7 this depth is unfortunately the last measured point. If deeper measurements were available in this borehole, the gradient may be less. The gradient in BH # 7 is definitely higher than BH # 3 but expecting a 107.5 °C/km in all the studies area is far from reality. Regarding presented interpretations, depth of the reservoir can be around one kilometer.

Regarding the result of the temperature measurements, a temperature map (for the depth of 50 m) is provided (Fig. 9). As this figure apparently shows, Abgarm thrust fault in the central part of the studied region paved the way for circulation of hot water stored in the depth. This modelling result is in close agreement results from the anomaly map. In Fig. 7, also Abgarm thrust fault passes through an important anomaly zone (A1). Therefore, the high temperature can be addressed as the source of the anomaly.

## Conclusion

In this study, all available data from Mahallat Region are exerted to present a deeper and more integrated view of the probable subsurface reservoir. Based on geological evidence, Jurassic shales (upper parts of Shemshak Formation) are the possible cap rocks of the reservoir. Mahallat warm springs fall into the bicarbonate water family regarding the geological features of the area and also geochemical components. Based on the cation geothermometry analyses, the probable temperature of geothermal reservoir is expected to be 90 °C. The gravity data are obtained from 380 stations and deciphered three main negative anomaly zones. One of them (A1) has an apparent relation to the geothermal activity in the study area. A1 anomaly zone is elongated parallel to Abgarm Fault with NE-SW strike. Geothermal gradient and thermal maps of the region are obtained based on the data collected from 7 gradient wells with the depths ranging from 50 to 100 m. The highest geothermal gradient is 107.5 °C/km, but the shallowness of the borehole raises doubt about the accuracy of value. Data coming out of another borehole (BH # 3) proposed a more reliable geothermal gradient (81.5 °C/km). Abgarm Fault acts as a conduit for deep hot water circulation in the borehole showing the surprisingly high geothermal gradient. Pervasive alteration zones, active tectonic, numerous hot springs and huge travertine exposures altogether bring the hope a prolific almost shallow subsurface hot reservoir exists.

## Data Availability

All used data can be published.
